# A novel Golgi protein (GOLPH2)-regulated oncolytic adenovirus exhibits potent antitumor efficacy in hepatocellular carcinoma

**DOI:** 10.18632/oncotarget.3769

**Published:** 2015-04-23

**Authors:** Yigang Wang, Tao Liu, Panpan Huang, Hongfang Zhao, Rong Zhang, Buyun Ma, Kan Chen, Fang Huang, Xiumei Zhou, Caixia Cui, Xinyuan Liu

**Affiliations:** ^1^ Xinyuan Institute of Medicine and Biotechnology, School of Life Sciences, Zhejiang Sci-Tech University, Hangzhou 310018, PR China; ^2^ Institute of Biochemistry and Cell Biology, Shanghai Institutes for Biological Sciences, Chinese Academy of Sciences, Shanghai 200031, PR China; ^3^ Otorhinolaryngology Head and Neck Surgery, The Affiliated Hospital of Hangzhou Normal University, Hangzhou 310015, PR China; ^4^ School of Public Health, Zhejiang University, Hangzhou 310058, China

**Keywords:** GOLPH2, hepatocellular carcinoma, oncolytic adenovirus, anticancer capacity

## Abstract

Golgi apparatus is the organelle mainly functioning as protein processing and secretion. GOLPH2 is a resident Golgi glycoprotein, usually called GP73. Recent data displayed that GOLPH2 is a superb hepatocellular carcinoma (HCC) marker candidate, and even its specificity is better than liver cancer marker AFP. Oncolytic adenoviruses are broadly used for targeting cancer therapy due to their selective tumor-killing effect. However, it was reported that traditionally oncolytic adenovirus lack the HCC specificity. In this study, a novel dual-regulated oncolytic adenovirus GD55 targeting HCC was first constructed based on our cancer targeted gene-viral therapeutic strategy. To verify the targeting and effectiveness of GOLPH2-regulated oncolytic adenovirus GD55 in HCC, the anticancer capacity was investigated in HCC cell lines and animal model. The results proved that the novel GOLPH2-regulated GD55 conferred higher adenovirus replication and infectivity for liver cancer cells than oncolytic adenovirus ZD55. The GOLPH2-regulated GD55 exerted a significant grow-suppressing effect on HCC cells *in vitro* but little damage to normal liver cells. In animal experiment, antitumor effect of GD55 was more effective in HCC xenograft of nude mice than that of ZD55. Thus GOLPH2-regulated GD55 may be a promising oncolytic virus agent for future liver cancer treatment.

## INTRODUCTION

Human hepatocellular carcinoma (HCC) is one of the most common and morbidity cancers worldwide. In China, patients with liver disease such as chronic hepatitis are at the highest susceptibility for developing HCC, which accounts for 55% of new HCC cases worldwide [1]. Hepatitis B and C virus are the most risk factors for HCC, especially for the tight relationship between hepatitis C virus and high HCC incidence [2]. The traditional therapeutic methods for HCC mainly include surgery, chemotherapy and radiotherapy et al, which are feasible for patients who are in the early phase, but not for most patients with advanced stage of liver cancer [3]. Thus, the traditional therapeutic methods are difficult to acquire desired effect for advanced or metastasis cancer. It is urgent to explore the novel treatment strategies for improving HCC patients' survival and living quality.

Recently, oncolytic adenovirus represents a great promise drugs to treat human cancers. So far many targeted strategies based on oncolytic adenovirus were designed and showed potent antitumor activity in various preclinical studies [4–6]. Our previous designed cancer targeting gene-virotherapy strategy (CTGVT), which combined the superiority of gene therapy and virotherapy, will bring the new hope for tumor therapy [5, 7]. Based on CTGVT, the novel oncolytic adenovirus system, ZD55 (ZD55-gene), was engineered through deleting adenoviral E1B55-kD viral protein [8]. It was previously reported that the adenovirus mutant *dl1520* (also named ONYX-015) with E1B55-kD deletion could preferentially target and lyse p53-dysfunctinal tumor cells but not in the adjacent normal cells [9], however, further studies denied this view point and proved that the adenovirus mutant can enhance the viral mRNA late nuclear transport and oncolysis for tumor selectivity [10]. ZD55 system was similar with ONYX-015. It not only can selectively replicate in cancer cells and kill them, but carry exogenous antitumor gene [8]. Preclinical data showed that ZD55-gene exhibited significant antitumor effect in multiple types of cancer models whether in tumor cell lines or in mice models through the oncolytic action of virus itself and increased expression level of the carried antitumor gene [4, 11, 12].

However, ZD55 lacks the targeting ability for specific tumor type such as liver cancer. Thus, to improve the specific killing effect of oncolytic adenovirus on one type of cancer, one common strategy to design oncolytic adenoviruses is to use cancer or tissue-specific promoter to control the expression of viral essential gene for replication, which is the transcriptional targeted strategy [13, 14]. It causes the viral gene selectively expression in tumor cells, then the virus could only replicate in and kill tumor cells [7, 15].

Besides advanced therapeutic strategy for HCC, more important factor for improving the cure rate of HCC patients is early diagnosis. Fortunately, the current early diagnostic technologies were greatly improved by the diversified serum marker, image modalities, and histologic detection, which led to the outstanding prognosis [16]. GOLPH2, a Golgi membrane glycoprotein GP73, is one of glycoprotein discovered in recent years. Many results demonstrated that GP73 is an excellent marker for HCC diagnosis, and its sensitivity and specificity are better compared with the common liver cancer marker α fetoprotein (AFP), which reach 75% and 97% separately, while 58% and 85% for AFP [17–19]. In previous study, the tumor-targeting gene-viral therapy was performed by oncolytic adenovirus-mediated the transgene gene expression regulating by AFP promoter and proved certain efficacies in HCC model [20, 21]. Due to the outstanding character of GOLPH2, we attempt to identify the liver cancer targeting and therapeutic efficiency of GOLPH2-regulating oncolytic adenovirus for cancer gene-viral therapy. The novel GOLPH2-regulated oncolytic adenovirus GD55 was first designed, in which endogenous E1A promoter was replaced by GOLPH2 promoter to regulate E1B- 55kD- deleted ZD55. It is unreported in the present studies. Meanwhile, we also constructed the adenovirus GD55-EGFP carried green fluorescent protein (EGFP). The experimental results *in vitro* showed that the GD55 has the better specificity of antitumor proliferation ability than that of ZD55, and exhibits the targeting antitumor effect in HCC cells with the lesser side-effect to liver normal cells. Further animal experiments *in vivo* showed that GD55 has good suppression effect on liver cancer growth in xenografted HCC mice.

In conclusion, the study has successfully screened the specific GOLPH2 promoter core region for HCC, and first constructed oncolytic adenovirus vector GD55 for targeting HCC. The preliminary results indicated that GD55 has excellent liver cancer specific and acts as the candidate of the individual targeting cancer gene-viral therapy for HCC patients, which lay on the foundation for future clinical liver cancer individual therapy.

## RESULTS

### Identification of GOLPH2 promoter and its high activity in liver cancer cells

**Figure 1:**
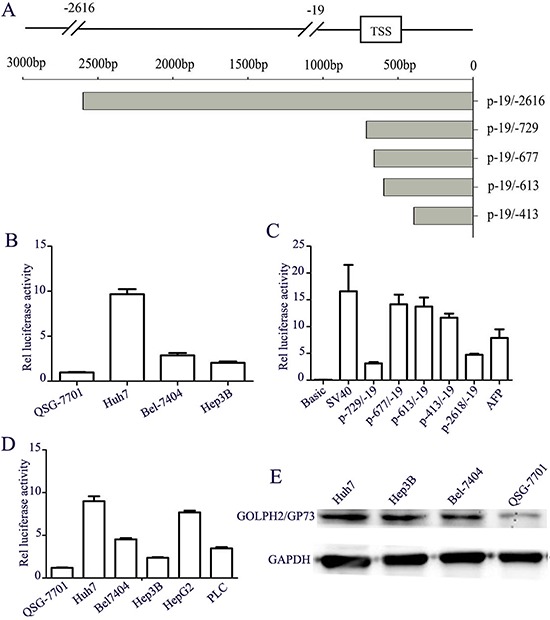
Analysis of GOLPH2 promoter activity and its expression in HCC cell lines **A.** The diagram pattern of human GOLPH2 promoter and its different truncations. **B.** Activity detection of human *GOLPH2* promoter with F-2618/-19. Three hepatocellular carcinoma cell lines Huh7, Bel-7404, Hep3B and normal liver cell line QSG-7701 were transiently transfected with pGL3-L1 (large *GOLPH2* promoter fragment F-2618/-19) and pRL2 was co-transfected as an internal control. Luciferase activities were calculated in relation to background activity of pGL3-basic. **C.** Truncations analysis of *GOLPH2* promoter. A series of promoter truncations are represented with different numbers and their Luciferase activities are shown relative to pGL3-basic activities in Huh7 cells. **D.** The activity of the 659 bp truncation promoter p-677/-19 was detected in five HCC cell lines and normal liver cells according to the above methods. **E.** Expression of GOLPH2 protein was detected by western blotting analysis in three HCC cell lines and normal liver cell line.

The 2.6 kb fragment upstream of GOLPH2 gene was first cloned into pGL3-basic named by p-2618/-19 by Dr. Peng, which indicated higher fluorescent intensity compared with control sequence in the EGFP reporter construct, and exhibited potent promoter activity in transient transfection assays [22]. We first detected the activity of long GOLPH2 promoter p-2618/-19 in liver normal epithelial cell QSG-7701 and hepatocarcinoma cell lines Huh7, Bel-7404, Hep3B with luciferase reporter assay. It was verified that all the hepatocarcinoma cell lines showed significantly higher GOLPH2 activity despite some variation compared with the normal cell line (Figure 1B). Among them, Huh7 cells displayed the highest GOLPH2 promoter activity. To map the minimal core promoter region and avoid potential regulatory inhibitory regions in 2.6 kb GOLPH2 promoter, we generated a series of promoter deletion truncations by PCR (Figure 1A) and subcloned into pGL3-basic, then measured their transcriptional activity in Huh7 cells. As shown in Figure 1C, the 659 bp truncation of the minimal core promoter p-677/-19, spanned from -677 to -19 bp, exerted the highest promoter activity. Subsequently, we measured the activity of the 659 bp truncation in various HCC lines, and found that the promoter activity is obviously higher in HCC lines than that of normal cells (Figure 1D). The HCC cell-specific expression of GOLPH2 protein was detected by western blotting analysis (Figure 1E). These results proved that the GOLPH2 promoter could be used to regulate the oncolytic adenovirus and drive the expression of exogenous genes specifically in hepatocarcinoma cells.

### Detection of GOLPH2 expression in HCC specimens

**Figure 2:**
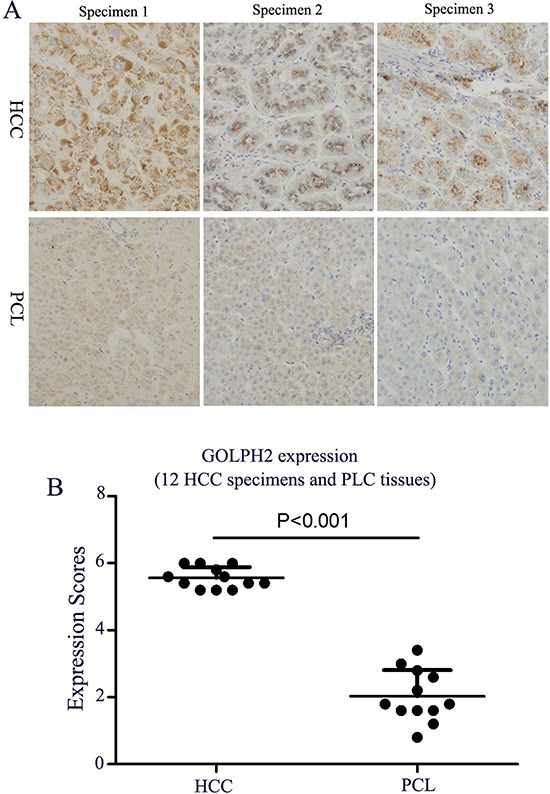
A. Identifcation of GOLPH2 expression in HCC specimens. HCC specimens and paracancerous liver (PCL) tissues collected from 12 HCC patients were fixed in 10% formalin prior to perform standard paraffn-embedded sections, and the expression of GOLPH2 was detected by the immunohistochemistry analysis **A.** The GOLPH2 expression was observed with microscope (original magnifcation: × 200). **B.** The expression scores of GOLPH2 in HCC and PCL tissues. The five medium-power fields in 20 × objective lens in each specimen were selected and the number of GOLPH2 positive cells was counted. The scores were defined by counting the stained cell ratio and the stained intensity from 0 to 6 scores.

The HCC specimens and the paracancerous liver (PCL) tissues were collected from 12 patients after undergoing clinical operation, and GOLPH2 expression was detected by immunohistochemistry analysis. The expression of GOLPH2 was positive in all HCC specimens, including strongly positive, moderately, and weakly positive (Figure 2A). Nevertheless, GOLPH2 expression in the PCL tissues was significantly weakened compared with HCC tissues, further indicating the liver cancer specificity of GOLPH2 expression.

### Construction of GOLPH2-regulated oncolytic adenovirus GD55 and its replication in hepatocarcinoma cells

**Figure 3:**
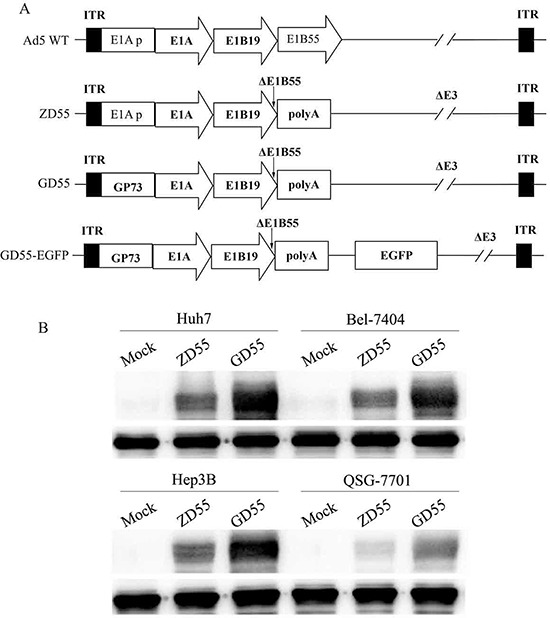
Characterization of GOLPH2 promoter-regulated oncolytic adenovirus GD55 **A.** Schematic structure of recombinant oncolytic adenovirus was compared to the wild type adenovirus (Ad 5 WT). GD55 was designed with E1A promoter replaced by *GOLPH2* core promoter and E1B 55kDa gene deletion, which obtains a dual-regulated oncolytic adenovirus propagating only in cancer cells and lysing them while sparing normal cells. ITR, inverted terminal repeat. **B.** Specific E1A expression in various cell lines. Cells were infected with ZD55 or GD55 at an MOI of 5 for 48 hrs. Non-infected cells were acted as a control. Lysates from different treated cells were subjected to western blot assay with anti-E1A antibody as described in Materials and Methods.

Human GOLPH2 promoter is active in liver cancer cells, but low in normal liver cells (Figure 1), which makes GOLPH2 promoter a promising candidate for designing HCC-specific oncolytic adenoviruses. Here, we constructed a cancer-targeted dual-regulated oncolytic adenovirus GD55, which characterizes with the replacement of the normal E1A transcription regulatory elements using 659 bp truncation of GOLPH2 core promoter, and the deletion of E1B 55kDa gene. The genome of the recombinant adenoviruses was depicted in figure 3A. In GD55, E1B 55kDa gene was removed and similar with oncolytic adenovirus ONYX-015, which was reported to enhance the viral mRNA late nuclear transport and oncolysis [10].

The essential of the oncolytic virus is able to selectively replicate in tumor cells and kill them. Thus, efficient viral replication and high progeny production can greatly contribute to the antitumor ability of oncolytic adenoviruses. Previously, we constructed the oncolytic adenovirus ZD55 with E1B 55kDa gene deletion, and proved its potent replication ability and antitumor effect *in vitro* and *in vivo* [8]. To compare the replication ability between dual-regulated virus GD55 and ZD55, liver cancer cells (Huh7, BEL7404, and Hep3B) and normal cells QSG-7701 were infected by indicated GD55 and ZD55. The results showed that higher expression of E1A in all GD55-infected liver cancer cells than that of normal liver cells (Figure 3B).

**Figure 4:**
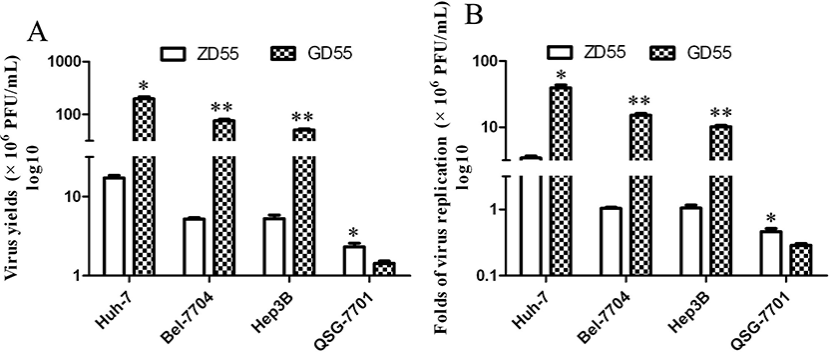
Replication of GD55 and ZD55 in hepatocarcinoma cells GD55 and ZD55 at an MOI of 5 were used to infect hepatocarcinoma cells and normal liver cells to evaluate their ability to produce viral progeny. **A.** Viral progeny were quantified with the TCID50 method in infected-hepatocarcinoma cancer cells. **B.** Fold replication values of oncolytic adenoviruses in infected-hepatocarcinoma cancer cells were calculated by the formula: Fold replication = titer of viral progeny at 48 hrs after infection/titer of viral progeny at infection. Results are the average of two independent experiments.

Further, production of infective viral progeny was quantified by the TCID_50_ method in ZD55- or GD55-infected liver cancer cells. As shown in Figure 4A, liver cancer cells infected with GD55 produced higher titers of viral progenies than that of normal liver cells, and the titer of viral progenies in GD55-infected liver cancer cells is 10-time high than in the ZD55-infected cells. Interestingly, GOLPH2-regulated GD55 has higher viral replication efficiency than ZD55 (Figure 4B). Thus, the novel dual-targeted virus GD55 replicated more efficiently and produced greater amounts of progeny viruses than ZD55 in liver cancer cells.

### GD55-mediated EGFP expression in liver cancer cells

**Figure 5:**
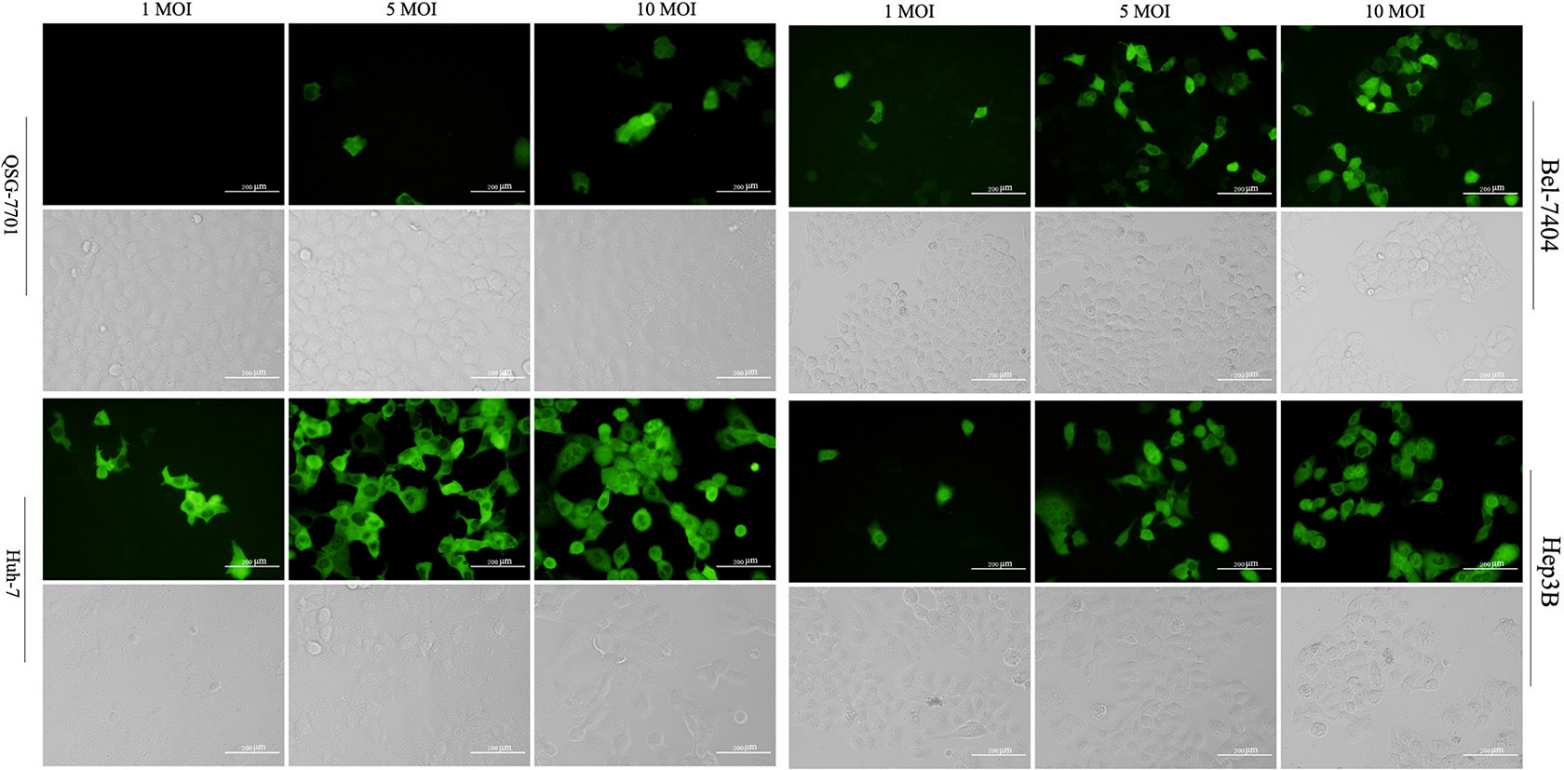
Tumor-specific EGFP expression driven by the GOLPH2 promoter The normal cell line QSG-7701 and three hepatocarcinoma cell lines Huh7, Bel-7404, Hep3B were infected with GD55-EGFP at an indicated MOI (1, 5, and 10), respectively, and the green fluorescent cells were observed under fluorescence microscopy after 48 hrs. The corresponding cell morphology to fluorescent cells was photographed in light field, which showed the death of hepatocarcinoma cells treated with GD55.

In consideration of the specificity of E1A expression mediated by GD55, we also evaluate its ability as a vehicle for exogenous gene transfer. We constructed the recombinant virus GD55-EGFP and observed the tumor targeting ability of dual-regulated oncolytic adenovirus through specific EGFP expression in HCC cell line infected with SD55-EGFP, which was dose-dependent ways in 48 hr postinfection (Figure 5). Meanwhile, few QSG-7701 normal cells express green fluorescence in same infection. Therefore, the corresponding bright field showed the obvious appearance of cytopathic effect such cell rounding and detachment, signifying that GD55-EGFP caused more cell death in liver cancer cells, but had no significant cytotoxicity to normal cells. These findings indicated that the novel dual-regulated oncolytic adenovirus GD55 system effectively mediated the tumor-specific transgene expression and exerted the tumor cell cytotoxicity, but less in normal cells.

### Tumor-killing effect of GOLPH2-regulated GD55 *in vitro*

**Figure 6:**
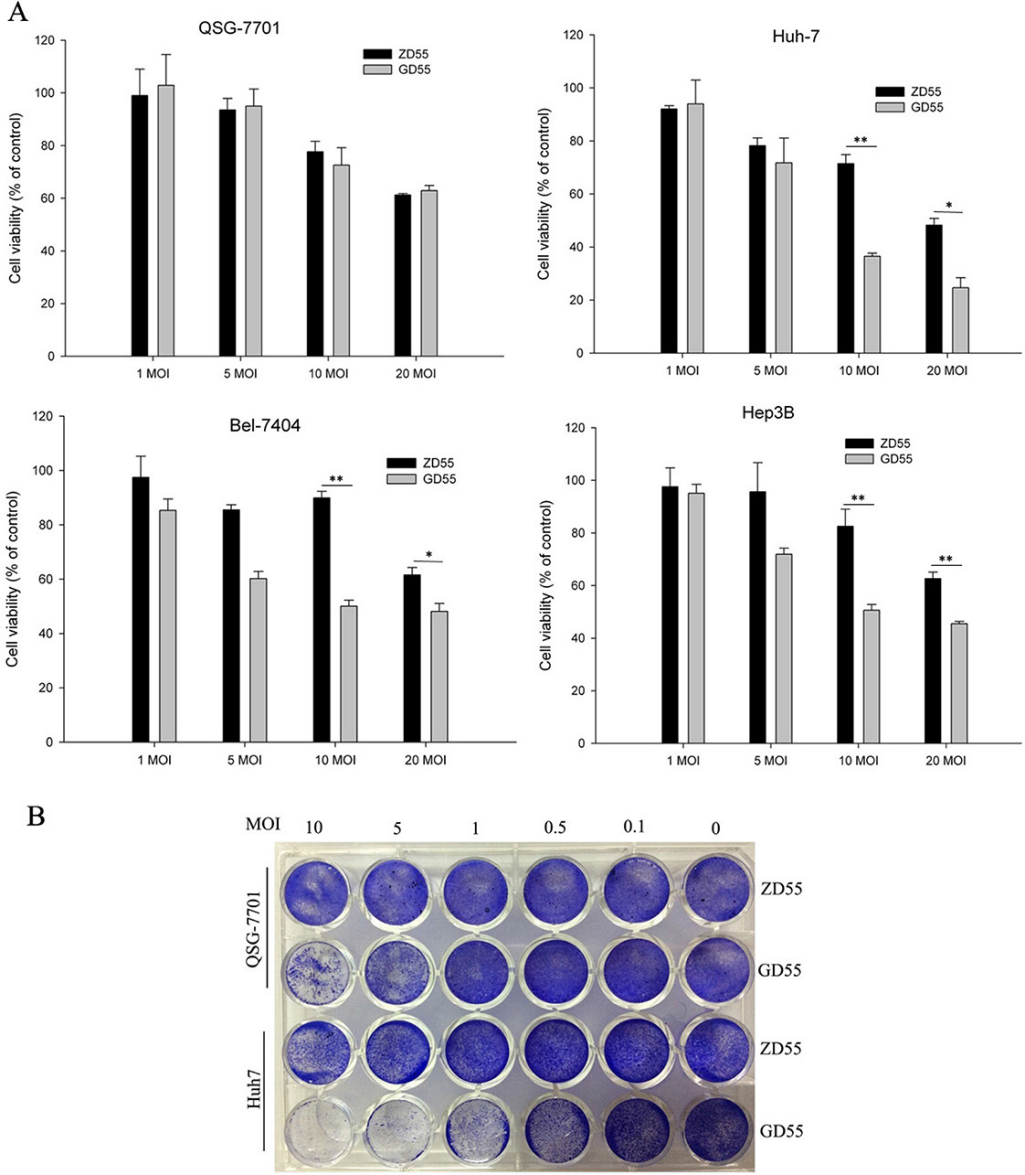
Cytotoxicity of GOLPH2 promoter-regulated oncolytic adenovirus for hepatocarcinoma cancer cells **A.** Survival rate of various cell lines *in vitro*. The HCC cell lines Huh7, Bel-7404, and Hep3B and normal liver cell lines QSG-7701 were infected with GD55 or ZD55 at MOIs of 1, 5, 10, and 20. After 72 hrs, the cell survival rate was measured by MTT assay. The results are presented as the mean ± SD (*n* = 3) and are expressed as the percentage relative to mock-treated control cells. **B.** Tumor-selective cytopathic effect of GD55 and ZD55. HCC Huh7 cells and normal QSG-7701 cells were infected with the oncolytic adenoviruses at the indicated MOIs. Two days later, cells were stained with crystal violet and cytopathic effect was shown with photograph.

Further tumor-suppressing activity of the GOLPH2-regulated GD55 was investigated *in vitro* by MTT analysis. Three liver cancer cell lines and normal cell line QSG-7701 were infected with GD55 and ZD55 at various MOIs. The data of cell viability showed that GD55 had a higher antitumor capacity for hepatocarcinoma cells than that of ZD55 at a dose-dependent manner (Figure 6A). The cell growth suppressive rate reached about 75.31% in Huh7 cells infected by GD55. However, the novel oncolytic adenovirus GD55 caused slight cytotoxicity in normal liver cells. To further monitor the cytotoxicity of GD55, liver cancer Huh7 and normal QSG-7701 cells were infected with GD55 or ZD55 at various MOIs. After 2 days, the cytopathic effect was detected through staining adherent cells with crystal violet. The results showed that GD55 induced the stronger cytotoxicity in Huh7 cells compared with ZD55, while GD55 led to about 20-fold attenuation in killing QSG-7701 than in Huh7 cells (Figure 6B). Similar results were obtained from other liver cancer cell lines (data not shown).

**Figure 7:**
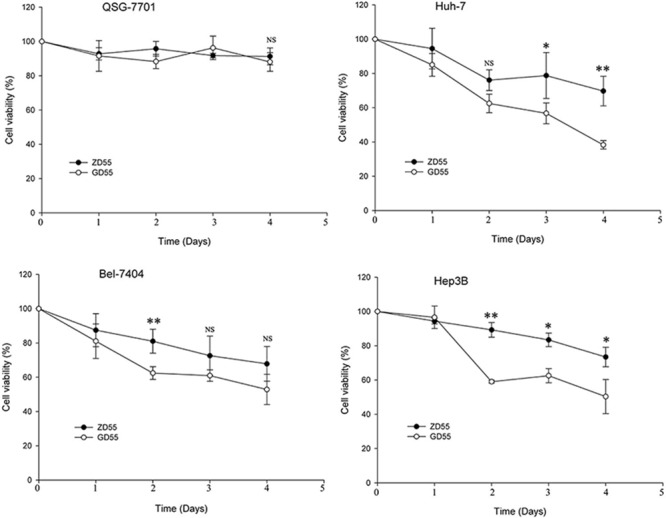
Cell viability of time-dependent by GOLPH2 promoter-regulated oncolytic adenovirus GD55 Three HCC cell lines (Huh7, Bel-7404, and Hep3B) and normal cell line QSG-7701 were infected with GD55 or ZD55d at a MOI of 5. On the day 1, 2, 3, and 4 postinfection, cell viability was measured with MTT assay. Results are presented as mean ± SD (*n* = 3) and are expressed as the percentage relative to mock-treated control cells.

The kinetics of cytotoxicity induced by GD55 also was evaluated in liver cancer cells and normal cells. As shown in Figure 7, the cytotoxicity effect of GD55 on the liver cancer cell lines was much more obvious than that of ZD55 with a time-dependent manner. There was little effect on normal liver cells infected by either GD55 or ZD55. Taken together, our data demonstrated that GOLPH2-regulated oncolytic adenovirus GD55 can eliminate liver cancer cells more effectively than common oncolytic adenovirus ZD55 *in vitro*.

### Antitumoral efficacy of GOLPH2-regulated GD55 for liver cancer in an animal model

**Figure 8:**
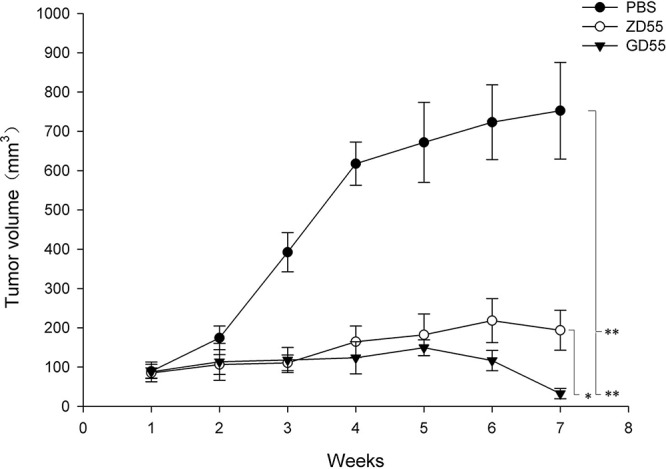
Antitumor efficacy of GD55 for hepatocarcinoma xenografts in nude mice Huh7 liver cancer cells were subcutaneously inoculated into female BALB/c nude mice at 5 × 10^6^ cells per mouse to tumor xenografts. When tumor volume reached 80–120 mm^3^, the mice were divided into three groups (*n* = 8) randomly and infected with 2 × 10^8^ pfu ZD55, GD55, or PBS every other day for repeated three times. The tumor volumes were monitored by periodic measurement. Tumor volume **(V)** was calculated according the formula: V (mm^3^) = length (mm) × width (mm)^2^/2.

Our *in vitro* experiments showed that GD55 could kill liver cancer cells. Further, we investigated the tumor-suppressing capacity of GD55 for liver cancer cells in nude mice. Figure 8 showed that tumor volume of different treatments of PBS, ZD55, and GD55. The data indicated that GD55 significantly and completely inhibited the growth of Huh7 cancer xenograft, and the antitumor effect is comparable with ZD55, even ZD55 also exerted the excellent suppression effect of tumor growth. Moreover, the tumor volume of mice was 752 mm^3^ and 194 mm^3^ at 7 weeks after treatment with PBS and ZD55 mice, respectively, while tumor size in GD55-treated mice was greatly reduced. It was noteworthy that the tumor xenograft in three of eight mice completely disappeared in GD55-treated group in the seventh week.

**Figure 9:**
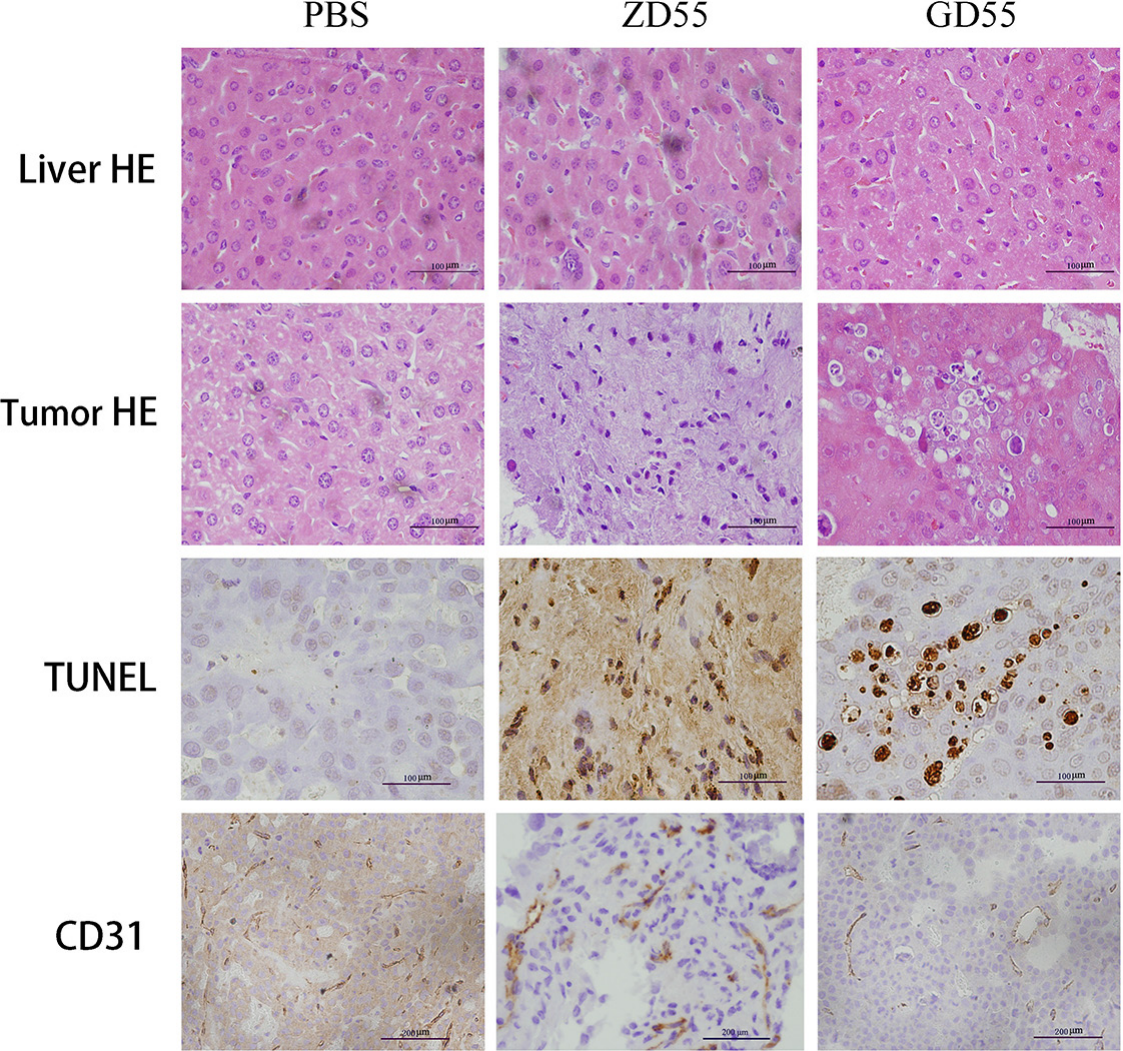
GD55 induced cell death *in vivo* Histological analysis of tumor sections in GD55, ZD55, and PBS groups for Huh7 tumor xenograft. The upper two rows are hematoxylin and eosin staining analysis for animal liver and tumor tissues, indicating that hepatotoxicity or cell necrotic area in tumors. The third row is TUNEL assay for detecting apoptotic cells in tumor tissues. GD55 induced more apoptosis of tumor cells. The brown color represents the apoptotic cells. The bottom row showed the immunohistochemical staining for vessel marker CD31, indicating that vessel growth in tumors treated with GD55 was significantly suppressed compared with the control-treated groups. Original magnification: × 200.

**Figure 10:**
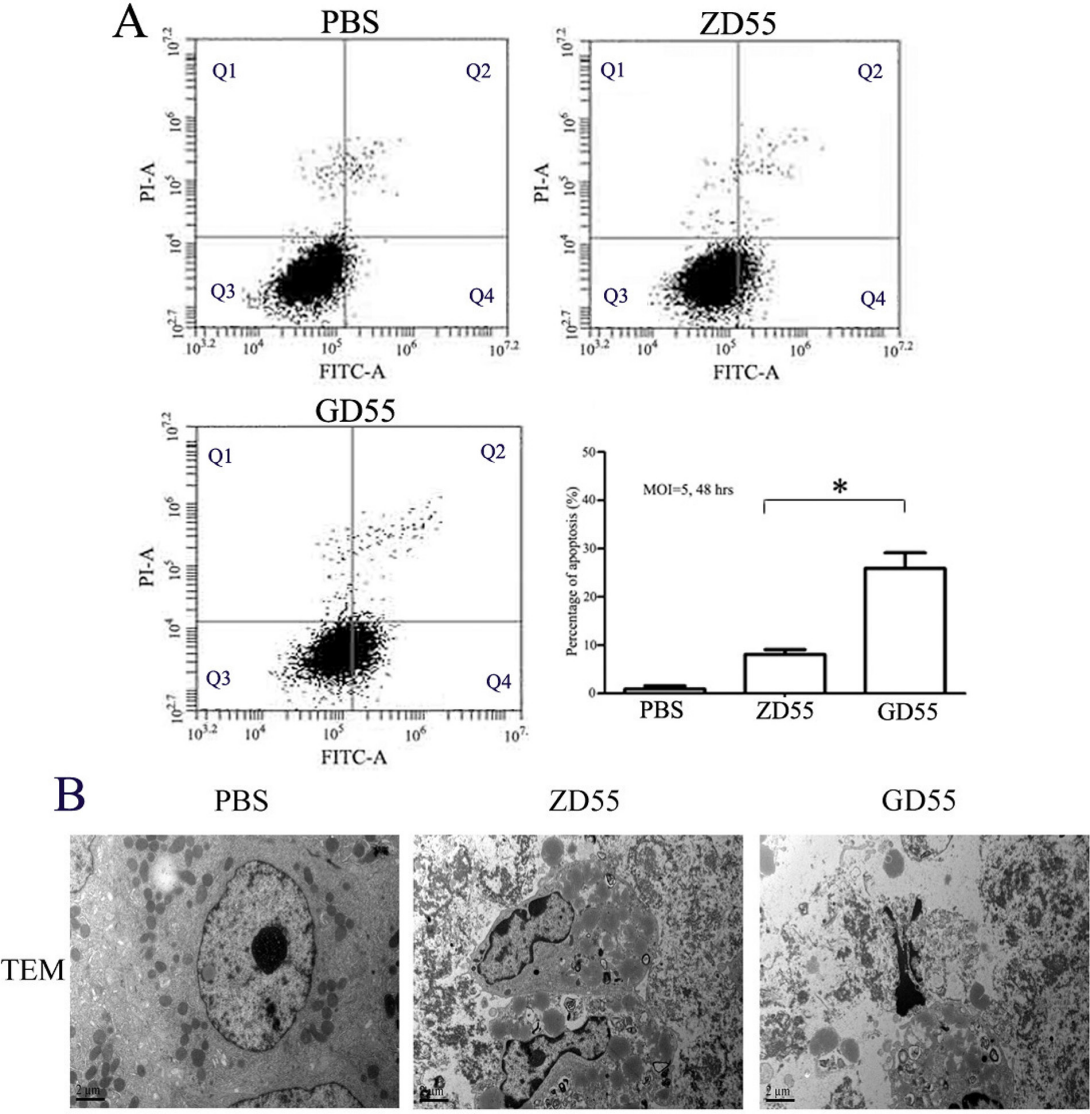
Apoptotic inducement in Huh7 cells by GD55 **A.** Annexin V binding assay. The Huh7 cells were infected with PBS, ZD55 or GD55 at an MOI of 5. 48 h later, the cells were stained with annexin V-FITC immediately followed by flow cytometry for apoptosis assay. Each value represents the mean of three wells. * < 0.01. **B.** Morphological observation of tumor tissue for cell apoptosis by TEM analysis. The obvious apoptosis was detected in tumors treated with GD55, secondly ZD55 than PBS treatments, such as nuclear collapse, appearance of nucleus deformation, and chromatin condensed in lumps et al.

The histo-pathological test by Hematoxylin and eosin staining showed that GD55 caused more profound cell death and symptoms of necrosis in tumor mass than ZD55, and that GD55 didn't lead to any obvious damage to liver tissue, which is essentially same to PBS-treated group (Figure 9). TUNEL assay indicated that GD55 treatment induced more extensive apoptosis in tumor tissue than in ZD55 or PBS treatment. IHC staining for CD31 also showed that vessel growth was significantly suppressed by GD55 treatment compared with ZD55 or PBS treatment, suggesting the antiangiogenesis effect of GD55. Further, we detected tumor cell apoptosis induced by GD55 with flow cytometric analysis. The percentage of apoptotic cells was determined by annexin V staining. As shown in Figure 10A, the Huh-7 cells infected with GD55 showed much higher percentage of cell apoptosis (25.9%), whereas, the lower percentage of cell apoptosis was observed in the liver cancer cells treated with ZD55 (8.05%) or PBS (1.0%) respectively. Morphologic observation of tumor tissue was also detected by TEM analysis (Figure 10B). The significant apoptosis was observed in tumor tissue treated with GD55, followed by ZD55 than PBS, as the characteristic of apoptosis, including nuclear collapse, the appearance of nucleus deformation and condensed chromatin in lumps at the inner side of nuclear envelope. The *in vivo* results suggested that an increased tumor-suppressing efficacy of GOLPH2-regulated GD55 for liver cancer than traditional oncolytic adenovirus ZD55, and the inhibitory effect on tumor growth was due to cell apoptosis and anti-angiogenesis.

## DISCUSSION

Recent many clinic trials using oncolytic virus highlights the value and hope for cancer-killing viruses. Oncolytic virus has emerged as one of the most prospective anticancer drugs because the engineered viruses can successfully tackle cancer cells without damaging normal tissue [15]. Several excellent genetically modified oncolytic virus including vaccine virus JX-594, herpes simplex virus OncoVEX-GM-CSF, adenovirus H101, and reovirus Reolysin have been successfully used to treat various cancers in clinic and obtained specific cancer-killing effect [14]. In addition, oncolytic virus can induce the immune response against the tumor through releasing antigens when virus attacked cancer cells [23].

Decade ago, we first put forward to the cancer targeting gene-viral therapy strategy based on the combination of oncolytic virus therapy and gene therapy [11], and constructed the genetically modified adenvirus ZD55, showing the characteristic of cancer-killing virus [8]. But it is lack of specificity for some kind of cancer. In this study, we described a novel oncolytic adenovirus named GD55 based on ZD55, in which GOLPH2 promoter was used to restrict the E1A expression in liver cancer cells. Given that numerous papers published showed the GOLPH2 is a valuable diagnostic marker for hepatocellular carcinoma, GD55 could be a powerful anticancer agent to liver cancer from epithelium.

Previous studies indicated high expression of GOLPH2 specifically in human hepatocellular carcinoma cells [24]. In consistent with them, our experiments proved that liver cancer cells tested, including Huh7, Bel-7404, and Hep3B, have higher GOLPH2 promoter activity than normal cells (Figure 1). GOLPH2-regulated GD55 induced more efficient adenovirus replication both in E1A expression and proliferation of virus progeny in liver cancer cells than that of E1B-55kDa deleted oncolytic adenovirus ZD55. These results may account for the efficient infection of GOLPH2-regulated GD55 for hepatocellular carcinoma in this study.

In view of the high expression of GOLPH2 in liver cancer cells, we next observed the infective efficiency of GOLPH2-regulated adenoviral vector to liver cancer cells. There a significantly higher expression of EGFP gene in GD55-EGFP-infected liver cancer cells than in normal cells, indicating that GOLPH2-regulated adenoviral vector has a potent specifically infectivity for liver cancer cells. On top of this, just like ZD55′s [8]and BioVex's [25] products, the liver cancer-targeted adenoviral vector GD55 can also be armed with a panel of anti-cancer genes which would have the potential for treating HCC, such as GM-CSF that enhance the body's immune response against tumors, TRAIL or IL-24 for tumor inhibition and apoptosis, endostatin for anti-angiogensis, and antitumoral microRNA, and prodrug activating enzyme et al. We expected that GD55 carrying anti-cancer genes will obtain the synergy anticancer capacity for further liver cancer therapy.

Simultaneously, our results showed that GOLPH2-regulated GD55 is more sensitive to liver cancer cells and exerted much more efficiently antitumor effect than that of simple oncolytic virus ZD55. Accordingly, animal experiments also confirmed that GOLPH2-regulated GD55 had an increased inhibiting ability of tumor growth for liver cancer cells in nude mice xenografts. Preliminary data showed that treatment of GOLPH2-regulated GD55 induced more apoptosis of tumor cells and on tumor tissues, which is consistent with studies on some other types of cancer-targeting oncolytic adenovirus [26], although the underlying detailed mechanism is still unknown.

Besides, there are still some challenges that need to handle during the clinical trials of cancer targeting gene-viral therapy. Most of all, the immune system can be a double-edged sword, prone to attacking the cancer tissues as well as the oncolytic viruses [27]. Thus, how to avoid immune response to viruses is an important issue, such as transcriptional targeting strategy using cancer-specific promoter to restrict viral replication in cancer cells. This made the modified viruses to infect efficiently the tumor microenvironment and lead to prolong anticancer effect. Another hurdle is the generation of the drug-resistance to oncolytic viruses [28]. It is inspiring that researchers achieved the significant therapeutic efficacy through oncolytic viruses joining chemotherapy and radiotherapy in the arsenal of cancer treatments [15, 29].

Although mostly GOLPH2 be reported as a novel marker for hepatocellular carcinoma and chronic hepatitis B or C, there are still some attentions. Figure 2 showed the differential expression levels of GOLPH2 in HCC patients, which implied that potential anticancer effect of GD55 on various types of HCC patients will vary with different activity of GOLPH2. A subset of patients with high GOLPH2 expression will respond to GD55. Thus it is essential to detect the GOLPH2 expression in HCC patients before using the GD55. Moreover, there are still some reports that GOLPH2 as a novel promising tissue biomarker for prostate cancer [30], gastric cancer [31], pancreatic cancer, AIDS progression [32]. This would enlarge the applied range of GOLPH2-regulated GD55 and implies for efficient gene-viral therapeutic effect to other cancers.

In summary, we have successfully engineered oncolytic adenovirus GD55 regulated by GOLPH2 promoter, and have proved that use of GOLPH2 promoter can restrict the GD55 replication in the liver cancer cells. The GOLPH2-regulated GD55 efficiently inhibit the growth of liver cancer cells both *in vitro* and *in vivo*. With the progress of GOLPH2 as a promising marker for hepatocellular carcinoma, the novel dual-regulated oncolytic virus GD55 can become an effective anticancer agent candidate for future therapy of human hepatocellular carcinoma.

## MATERIALS AND METHODS

### Cell lines and culture

The HEK293 cell line was obtained from were purchased from ATCC (American Tissue Culture Collection, Rockville, MD). Human normal liver cell line QSG-7701, Human hepatocellular carcinoma (HCC) cell lines Huh7, BEL-7404, and Hep3B were purchased from Shanghai Cell Collection (Shanghai, China). The HEK293 and QSG-7701 cells were cultured in phenol red-free Dulbecco's modified Eagle's medium (DMEM; Gibco BRL, Grand Island, USA) supplemented with 10% fetal bovine serum (FBS, Gibco BRL). The Huh7, BEL-7404, and Hep3B cells were grown in DMEM (GIBCO BRL) supplemented with 10% FBS. All cells were incubated at 37°C in a humidified air atmosphere with 5% CO_2_.

### Construction of human GOLPH2 promoter truncations and luciferase reporter assay

A 2597 bp genomic fragment (−19 to −2618 bp) extracted from genomic DNA of Hela cells was inserted into pGL3-basic Firefly luciferase reporter vector (Promega), which was kindly presented from Dr. Tao Peng from GIBH, CAS [22]. Promoter deletions designated as p-2618/-19, p-729/-19, p-677/-19, p-613/-19, p-413/-19 were generated by PCR and subcloned into pGL3-basic plasmids.

The luciferase reporter assay for various GOLPH2 promoter truncations was done according to the Dual-glo luciferase assay kit (Promega). Briefly, cells were seeded in 96-well culture plate overnight before transfection. GOLPH2 promoter plasmids (100 ng/well) were transfected into normal liver cell line QSG-7701, HCC cell lines Huh7, BEL-7404, or Hep3B with Lipo2000™ (Life Technology) together with pRL-TK (10 ng/well) as an internal control to normalize transfection efficiencies. After 36 h transfection, cells were harvested. The luciferase reporter assay was done according to the Dual-glo luciferase assay kit (Promega). All constructs were performed in triplicate and repeated three times. Results from one representative experiment were shown.

### Identification of GOLPH2 expression in HCC specimens

HCC specimens and paracancerous liver (PCL) tissues were obtained from the First Affiliated Hospital of Wannan Medical University by surgical resection in March 2014. The patients, average age 51 years old (39–72), did not received chemotherapy or radiotherapy prior to surgery. The resected specimens were diagnosed pathologically as HCC. The specimens of HCC and PCL tissues were fixed in 10% formalin prior to prepare the paraffin-embedded sections, and the expression of GOLPH2 was detected by immunohistochemistry with the mouse anti-human GP73 antibody at a dilute concentration of 1:50 and the avidin-biotin-peroxidase complex reagent and diaminobenzidine tetrahydrochloride (DAB) solution (Vector Laboratories, Burlingame, CA). The sections were then counterstained with hematoxylin. The result of expression scores was determined by scoring the stained cell ratio and the stained intensity for each specimen [27]. Briefly, the five medium-power fields (20 × objective lens) were selected and the number of positive cells was counted under microscope. The stained intensity scores were decided as follows: staining identical to the negative control was defined as 0, while yellow, brown, and tan staining was defined as 1, 2, and 3, respectively. The stained scores of positive cell ratio were decided as follows: the score was 0 when all cells were negatively stained, the scores were 1, 2, and 3, respectively, if the proportion of the positively stained cells was 1/3 or less, 1/3 to 2/3, and 2/3 or more.

### Recombinant adenovirus construction and production

The adenovirus shuttle vector pSD55, pCA13-EGFP, pXC2, and adenoviral packaging vector pAdeasy are conserved in our lab. The oncolytic adenovirus plasmid pZD55 with E1B55-kDa gene deletion and oncolytic adenovirus ZD55 were constructed by our group previously [8]. The human GOLPH2 promoter (GP73) from pGL3 was further subcloned into Xho I and SnaB I site of plasmid pXC2 by replacing endogenous E1A promoter to generate the pXC2-GOLPH2. Then, the fragment GOLPH2-E1A obtained from pXC2-GOLPH2 with Xho I and Xba I enzyme and was inserted into pSD55 after the same site to form the adenovirus shuttle plasmid pSD55-GOLPH2, the double-regulated virus plasmid. The reporter gene EGFP expression cassette cut from pCA13-EGFP by Bgl II was inserted into pSD55-GOLPH2 using the same site, and the pSD55-GOLPH2-EGFP was constructed. All constructs were confirmed by restriction enzyme digestion and DNA sequencing.

### Generation, identification, purification and titration of adenovirus

The oncolytic adenovirus GD55 and GD55-EGFP were generated by homologous recombination Escherichia coli strain, BJ-5183. Briefly, the shuttle plasmid pSD55-GOLPH2 or pSD55-GOLPH2-EGFP and adenovirus packaging backbone plasmid pAdeasy-1 were co-transfected into BJ-5183, and the recombinant adenovirus genome pGD55 or pGD55-EGFP was obtained. After the digestion with Pac I, pGD55 or pGD55-EGFP was transfected into HEK293 cells using Effectene transfection reagent (Qiagen, CA). After homologous recombination in HEK293 cells, the GOLPH2-regulated E1B 55kD gene deletion oncolytic adenovirus GD55 and GD55-EGFP containing enhanced GFP-expressing cassette were generated. Each recombinant adenovirus was isolated with plaque purification and amplified in HEK293 cells. The adenoviruses were harvested and purified by ultracentrifugation in a cesium chloride gradient. The virus titers were determined using the tissue culture 50% infectious dose (TCID50) method in HEK293 cells and calculated as plaque-forming units (pfu)/ml.

### Assay of reporter gene virus infection and viral progeny

Normal cells and liver cancer cells were seeded in 24-well plates at a density of 5 × 10^4^ cells. Then, cells were infected with GD55-EGFP at different MOI (multiplicity of infection) of 1, 5, 10. 48 hr later, EGFP expression was observed under an Olympus fluorescence microscope with Olympus camera DP70. To determine the viral progeny, cells were seeded in 6-well plates and infected with GD55 or GD55-EGFP at a MOI of 5, After 5 hr of infection, cell medium was removed. Cells were washed with phosphate-buffered saline (PBS), and then 2 ml of fresh medium was added. 48 hr later, cells and medium were collected, and virus supernatant was collected by three freeze-thawing cycles and centrifugation. Virus yield was determined by TCID50 assay in HEK293 cells according standard protocol.

### Cell viability assay

Various cells were seeded in 96-well plates at a density of 1 × 10^4^ cells/well and infected with viruses at the indicated MOIs and times. The cell viability rate was measured by the 3-(4, 5-dimethylthiazol-2-yl)-2, 5-diphenyltetrazolium bromide (MTT; Sigma Chemical Co., St. Louis, MO, USA) assay according the formula: cell survival=(absorbance value of infected cells-blank)/(absorbance value of uninfected control cells). Briefly, 20 μl MTT (5 mg/ml in PBS) was added into each well. 4 hr after cells incubated at 37°C, the mediums and MTT were removed, and 150 μl of Dimetyl Sulfoxide was added. The absorbance value at 595 nm was read with a DNA microplate Reader. Four replicate wells were done at each MOI, and every experiment was repeated three times.

### Cytopathic assay

Normal cells QSG-7701 and human HCC cells Huh-7 were seeded in a 24-well plate. When cells were grown to a subconfluent level, and infected with different adenoviruses at the indicated MOIs. After 96 hr incubation at 37°C, the medium was removed and cells were stained with 2% crystal violet in 20% methanol for 30 min. Then, the plates were lightly washed with tap water, naturally dried and documented as photographs.

### Flow cytometric analysis

The Huh-7 cells were trypsinized after different treatments, and washed once with complete medium. Aliquots of cells (5 × 10^5^) were resuspended in binding buffer and stained with fluorescein isothiocyanate (FITC)-labeled annexin V and propidium iodide (PI) (Beyotime Biotech., China) according to the manufacturer's instructions. A fluorescence-activated cell sorting (FACS; Becton Dickinson) assay was immediately performed after staining.

### Western blot analysis

Cells with different treatments were washed three times with ice-cold PBS, and lysed in buffer (50 mM Tris-HCl, pH8.0, 150 mM NaCl, 100 μg/ml PMSF, 1% TritonX-100) for 30 min at ice. Cells were collected and cell debris was removed by centrifugation. The protein concentration of cell lysates was measured by bicinchoninic acid (BCA) protein assay kit (Beyotime, China). Protein samples were diluted with sodium dodecyl sulfate/polyacrylamide gel electrophoresis (SDS-PAGE) loading buffer, and then separated by 10% SDS-PAGE and transferred onto 0.45 μm pore size nitrocellulose membranes (Millipore Corp., MA, USA). Then, nonspecific reactivity was blocked with 5% fat-free dry milk in Tween 20-containing Tris-HCL buffered saline overnight at 4°C. The membrane was incubated with primary antibody for E1A and GOLPH2 protein (Santa Cruz Biotech., USA). After incubation in the dark with IR Dye 800 conjugated IgG secondary antibodies (Rockland Inc., UK), immunodetection was performed by using an Odyssey infrared imaging system (LI-COR Biosciences Inc., Lincoln, NE, USA). The same membrane was then used for detecting the expression of GAPDH using primary antibody against human GAPDH (Santa Cruz Biotech., USA).

### Animal experiment

All procedures for animal experiments were followed according to the guide for regulations and standards of Experimental Animal of the U.S. Department of Agriculture and the National Institutes of Health. Female BALB/c nude mice of 4–5 weeks old were purchased from the Animal Research Committee of the Institute of Biochemistry and Cell Biology (Shanghai, China). Huh7 tumor xenografts were established by subcutaneously inoculating 5 × 10^6^ Huh7 cells (suspended in 100 μl PBS) into right flank of each nude mouse. When the tumors reached 80–120 mm^3^, mice were randomly divided into three groups (*n* = 8). The tumor xenografts were intratumorally administrated with 100 μl of PBS with or without 2 × 10^8^ pfu of ZD55 or GD55. The injections were repeated three times every other day, and a total dosage 6 × 10^8^ pfu of adenoviruses per mouse. Tumor growth was monitored by periodic measurement using calipers. Tumor volume (V) was calculated by the formula: V (mm^3^) = length (mm) × width (mm)^2^/2. Tumors were harvested on day 4 posttreatment for histopathological and transmission electron microcopy (TEM) analysis. Animals were sacrificed when the diameter of tumors reached 2 cm. In this experiment, no mice with tumor xenografts were observed to die from tumor loading.

### Immunohistochemistry and TUNEL assay

Tumor tissues were fixed in 4% formaldehyde, dehydrated with gradient ethanol, and embedded in paraffin wax. Tissue sections (5 μm) were then dewaxed and rehydrated, were stained with hematoxylin and eosin. For immunohistochemical (IHC) analysis, deparaffinized tumor sections were treated with rabbit monoclonal anti-CD31 antibodies at 1:500 dilutions. The slides were then washed with PBS and incubated with goat anti-rab IgG antibodies-HRP polymers with 1:100 dilution. The slides were washed with PBS and incubated with the avidin-biotin-peroxidase complex reagent (Vector Laboratories, Burlingame, CA) and detected with DAB solution. The sections were then counterstained with hematoxylin. For terminal deoxynucleotidyl transferase-mediated dUTP-bioth nick end labeling (TUNEL) assay, an in situ apoptosis detection kit (Sino-American Biotechnology Co., Luoyang, China) was used in tumor tissue sections. Briefly, after incubation with proteinase K, tumor sections were dewaxed and rehydrated. Then, endogenous peroxidase was blocked with 3% H_2_O_2_, and sections were incubated with equilibration buffer and terminal deoxynucleotidyl transferase (TdT) enzyme. The sections were then incubated with antidigoxigenin-peroxidase conjugate. Peroxidase activity was detected by the staining of DAB solution.

### TEM analysis

Tumor samples (1 mm^3^) were fixed in a phosphate-buffered mixture with 2.5% glutraldehyde for 2 hr, and rinsed by PBS 15 min for three times, followed by 1 hr of fixation with 1% osmium tetroxide. After rinsed in 0.1 M phosphate buffer, the tumor samples were subjected to a graded series of dewatering treatment with ethanol and propylene oxide, and then embedded in pure acetone with embedding buffer at a ratio of 1:2 at room temperature for 3 to 4 hours, followed by embedded in pure acetone with embedding buffer at different gradient of temperature and time. The sections were suffered to double-stain with uranyl acetate and lead citrate, examined and photographed with a JEOL 100CX transmission electron microscope.

### Statistical analysis

Absorbance value in the MTT assay and mean tumor volume are presented as mean ± SD. Statistical analysis was performed using a one-way analysis of variance (ANOVA) and compared at a given condition by two-tailed t test. The difference between data were considered to be statistically significant when *P* < 0.05 (*), to be very significant when *p* < 0.01 (**) and to be very much significant when *p* < 0.001.
